# Dermal Scaffolds in Complex Extremity Wound Reconstruction: Impact of Early Application on Grafting and Flap Requirements—A Systematic Review

**DOI:** 10.1111/wrr.70219

**Published:** 2026-10-04

**Authors:** Rania Darwish, Simon M. Kratzer, Deepak M. Kalaskar, Peter Butler, Suvashis Dash

**Affiliations:** ^1^ Division of Surgery & Interventional Science, Royal Free London NHS Foundation Trust University College London London UK; ^2^ Division of Surgery and Interventional Science, Department of Plastic and Reconstructive Surgery, Royal Free London NHS Foundation Trust University College London London UK; ^3^ Department of Plastic and Reconstructive Surgery All India Institute of Medical Sciences (AIIMS) New Delhi India

**Keywords:** burn injury, dermal scaffold, extremity wounds, flap reconstruction, skin graft, trauma reconstruction, wound healing

## Abstract

Complex extremity wounds from burns or trauma often involve exposed tendon or bone, making direct skin grafting unreliable and increasing the likelihood of flap reconstruction. This review evaluated whether early dermal scaffold application reduces subsequent surgical requirements. PubMed, Embase, the Cochrane Library and clinical trial registries were searched for studies published between 2016 and 2026. Of 409 records screened, 10 studies comprising 415 scaffold‐treated wounds in 414 patients were included. Outcomes were synthesised descriptively and stratified by aetiology; meta‐analysis was precluded by heterogeneity. Across nine non‐randomised studies (393 patients), 38 (9.7%) healed without grafting and 352 (89.6%) were grafted. Graft avoidance differed markedly by aetiology: 32 of 192 trauma patients (16.7%) versus 0 of 190 burn patients (0%). All trauma events arose from three studies of small, highly selected defects, whereas three further trauma cohorts using immediate or staged grafting reported none. In the single randomised trial, scaffold take succeeded in 5 of 22 patients (22.7%) and all were grafted, whereas the graft‐only comparator achieved complete coverage success, although scar quality was better with the scaffold (Vancouver Scar Scale 2.40 vs. 7.45, *p* < 0.001). No study contained a flap comparator, and certainty of evidence was very low for five of seven outcomes and low for two. Current evidence suggests early dermal scaffold application may reduce reconstructive burden in selected trauma wounds, whereas in burns scaffolds appear to enable staged grafting rather than replace it. Reconstructive downstaging is presented as an exploratory framework, not a validated outcome.

## 1 Introduction

Complex wounds of the upper and lower extremities remain among the most difficult problems in plastic and reconstructive surgery [1, 2]. These wounds commonly arise after trauma or burns and may involve exposed tendon, bone, cartilage or joint structures [3, 4, 5]. In such situations, conventional split‐thickness skin grafting is often inadequate because graft survival depends on a well‐vascularised wound bed [6, 7]. This limitation frequently shifts reconstruction towards local or free flap surgery [1, 6]. Although effective, flap reconstruction carries greater operative complexity, donor‐site morbidity, longer recovery and higher physiological burden, especially in medically frail patients or in wounds complicated by contamination, infection or compromised vascularity [1, 5, 8, 9].

Dermal scaffolds have therefore emerged as an intermediate reconstructive option [10]. Rather than simply covering the wound, they aim to generate a neo‐dermis or vascularised bed over exposed or poorly perfused structures, allowing subsequent grafting or, in selected cases, spontaneous epithelialisation [3, 11, 12, 13]. This review therefore asked not only whether wounds healed, but whether early scaffold use reduced the need for grafting, reduced its burden or was associated with avoidance of flap reconstruction.

The included studies span a broad range of technologies: synthetic NovoSorb Biodegradable Temporising Matrix (BTM) (PolyNovo Ltd., Port Melbourne, Australia) [3, 4, 14, 15], and biological or biosynthetic matrices including Integra‐type bilayers (Integra LifeSciences, Princeton, NJ, USA), MatriDerm (MedSkin Solutions Dr. Suwelack, Billerbeck, Germany), PELNAC (Gunze Ltd., Osaka, Japan), human acellular dermal matrix and DuraGen [6, 7, 16, 17]. These differ substantially in composition, architecture and intended role (Section 4.4, Table S2); dermal scaffolds are not a single intervention but a family of tools [18, 19]. A common question nonetheless remains: can they meaningfully reduce reconstructive burden in extremity wounds?

The evidence base remains uneven [19, 20]. Broader burn literature suggests dermal substitutes may improve scar quality while delaying early closure [19, 21], and methodological inconsistency persists [18, 19]. A review restricted to burn‐ and trauma‐related extremity wounds is therefore useful, particularly when organised around surgical decision‐making rather than scaffold brand.

### 1.1 Terminology

The terms dermal scaffold, dermal substitute, dermal regeneration template, artificial dermis and dermal matrix are used inconsistently and often interchangeably in the primary literature. For clarity and consistency, the umbrella term dermal scaffold is used throughout this review to denote any acellular biological, biosynthetic or synthetic construct applied to a wound bed with the intention of generating a vascularised neo‐dermis prior to, or instead of, definitive autologous coverage. Where a statement refers to a specific product, the proprietary name is used. Terms such as dermal regeneration template and dermal substitute appear only within direct descriptions of the source studies or product nomenclature.

## 2 Methods

This review was conducted in accordance with the PRISMA 2020 guidelines [22] and was registered with PROSPERO on 7 April 2026 (registration number CRD420261360486; record version 2.0, published 8 July 2026). The review was registered before data extraction and risk of bias assessment were undertaken. Deviations from the registered protocol are declared in full in Section 2.10 and Table S6. The search was conducted across PubMed, Embase and the Cochrane Library between 1 and 15 April 2026 and identified an initial yield of 446 records. Following deduplication, 409 records underwent title and abstract screening.

Clinical trial registries were also searched to identify ongoing, unpublished or recently completed studies. ClinicalTrials.gov and the WHO ICTRP were searched on 15 June 2026 using terms related to dermal scaffolds, dermal substitutes, dermal regeneration templates, NovoSorb BTM, Integra, MatriDerm, Pelnac, burn wounds, trauma wounds and extremity wounds. Registry records were screened against the same eligibility criteria as the database search. Several potentially relevant registry records were identified, including trials involving NovoSorb BTM, Integra, Pelnac and Glyaderm; however, no additional eligible completed studies with extractable outcome data were identified beyond those already captured through database searching.

A second reviewer (S.M.K.) independently performed full‐text screening, data extraction and risk of bias assessment for all included studies. Extracted variables, including study design, patient population, scaffold type, outcome classification and synthesis grouping, were verified against the original publications. Any disagreements were resolved through discussion with the senior authors, with consensus reached in all cases.

### 2.1 Rationale for the Search Period

The search was restricted to studies published between 2016 and 2026. This window was selected because it corresponds to the period in which the current generation of dermal scaffolds entered routine extremity practice: NovoSorb BTM was first reported in clinical series from 2016 onwards, MatriDerm received United States Food and Drug Administration approval in 2021 and contemporary protocols for staged scaffold‐to‐graft reconstruction, adjunctive negative pressure wound therapy and early debridement differ materially from those used in earlier decades. Restricting the window therefore improves the clinical applicability of the synthesis to present‐day practice.

The consequence is acknowledged: several historically important studies fall outside the window, including Burke and colleagues [10], Heimbach and colleagues [21] and Moiemen and colleagues [13]. These are cited as background but were not eligible for extraction. The review therefore characterises contemporary rather than complete evidence.

### 2.2 Research Question and PICO Framework

This review addressed whether early application of dermal scaffolds reduces subsequent surgical requirements in the management of complex extremity wounds resulting from trauma or burns. The question was structured according to the PICO framework. The population comprised patients with complex extremity wounds of the hand, foot, leg or arm caused by trauma, burns or combined mechanisms. The intervention was early application of a dermal scaffold as the primary reconstructive step. The comparison, where available, was standard reconstructive care such as split‐thickness skin grafting alone or immediate flap reconstruction. The outcome was a reduction in surgical requirements, conceptualised as complete avoidance of skin grafting, a reduction in graft size or thickness or avoidance of flap reconstruction.

### 2.3 Outcomes

The primary outcome was graft burden, operationalised across three prespecified categories: cancellation, defined as complete avoidance of skin grafting; sparing, defined as a reduction in graft size, thickness or donor‐site requirement; and flap avoidance, defined as the use of a dermal scaffold to enable a simpler, graft‐based reconstruction in a wound for which flap reconstruction was documented as the alternative.

Secondary outcomes, recorded where reported, included scaffold take rate, skin graft take rate, time to healing, complication rate, number of secondary procedures, treatment failure or reconstructive escalation, mortality, length of hospital stay, functional and scar‐related outcomes and cost.

### 2.4 Analysis Unit and Denominators

Included studies reported outcomes in different units, stated here explicitly rather than reconciled by assumption. Fulchignoni and colleagues reported 27 fingers in 26 patients [16] and Ling and colleagues 18 hands in 15 patients [23]; the remaining eight reported patients. The denominator used throughout is the unit in which each study reported its reconstructive outcomes, giving 415 scaffold‐treated wounds in 414 patients. The difference arises from Fulchignoni and colleagues, whose graft‐avoidance outcomes are per finger and cannot be re‐expressed per patient. For Ling and colleagues, whose reconstructive outcome was donor‐site related and reported per patient, the patient denominator is used.

Consequently 15 of the 38 graft‐avoidance events are finger‐level, and pooled proportions describe scaffold‐treated wounds rather than individual patients. The analysis unit for every study is given in Table 1.

**TABLE 1:** Characteristics and reconstructive outcomes of the included studies.

Study	Design	Aetiology/site	Scaffold	Analysis unit (*n*)	No graft *n* (%)	Grafted *n* (%)	Primary reconstructive role
Wu‐Fienberg 2021 [24]	Retrospective case series	Trauma (1/6 burn); upper extremity	NovoSorb BTM	6 patients	3 (50.0)	3 (50.0)	Graft avoidance in small defects
Fulchignoni 2022 [16]	Retrospective comparative	Trauma; fingertip	MatriDerm	27 fingers (26 patients)	15 (55.6)	12 (44.4)	Graft avoidance in defects < 2 cm^2^
Gonzalez 2022 [2]	Retrospective comparative	Trauma (96.8%); extremity, mainly forearm	Integra Meshed Bilayer Wound Matrix ± NPWT	109 patients	0 (0)	109 (100)	Graft‐enabling; NPWT improves take
Hones 2023 [1]	Retrospective case series	Mixed (trauma, iatrogenic, burn, DFU, tumour, NF); extremity	MatriDerm	11 patients	6 (54.5)	2 (18.2)	Graft avoidance in flap‐unsuitable patients
Jou and Chepla 2024 [15]	Retrospective comparative cost analysis	Trauma; upper extremity	NovoSorb BTM vs. Integra CCS	27 patients (18 BTM, 9 CCS)	14 (51.9)	13 (48.1)	Graft‐burden and cost reduction
Tapking 2024 [14]	Retrospective cohort	Burn 149/175 (85.1%); ≥ 10% TBSA	NovoSorb BTM	175 patients	0 (0)	175 (100)	Graft‐enabling at scale
Song 2024 [7]	Case series	Post‐traumatic/post‐surgical; lateral heel	Human ADM + STSG + NPWT	11 patients	0 (0)	11 (100)	Graft‐enabling; defect filling
Yadav 2024 [6]	Prospective interventional series	Trauma; extremity (2/12 scalp)	DuraGen + immediate SSG	12 patients (10 extremity)	0 (0)	12 (100)	Graft‐enabling over minor exposure
Ling 2024 [23]	Retrospective comparative cohort	Burn; whole hand dorsum	ADM + thin STSG vs. thick STSG	15 patients (18 hands)	0 (0)	15 (100)	Donor‐site burden reduction
Rady 2025 [17]	Randomised controlled trial	Burn; upper limb	PELNAC vs. graft only	22 patients (Pelnac arm)	0 (0)	22 (100)	Scar quality only; no graft reduction

*Note:* Percentages are calculated on the analysis unit stated. Hones 2023 additionally reported 3 treatment failures (27.3%), one of which required flap reconstruction.

Abbreviations: ADM, acellular dermal matrix; BTM, biodegradable temporising matrix; CCS, collagen‐chondroitin silicone; DFU, diabetic foot ulcer; NF, necrotising fasciitis; NPWT, negative pressure wound therapy; SSG, split skin graft; STSG, split‐thickness skin graft; TBSA, total body surface area.

### 2.5 Approach to Quantitative Synthesis

Formal meta‐analysis was not performed. Pooling effect estimates across the included studies would have been inappropriate for three reasons: the interventions comprised seven distinct scaffold products differing in composition, architecture and intended staging; only three studies contained any comparator group, and in two of these the comparator was an alternative scaffold or an adjunctive therapy rather than standard reconstruction; and outcome definitions were not standardised, with no consistent definition of graft avoidance, flap avoidance or scaffold take across studies.

Instead, a structured descriptive synthesis was undertaken. Raw event counts were extracted from each source and unweighted crude proportions calculated per study and per stratum. These are descriptive summaries, not pooled effect estimates: they carry no confidence intervals, are unadjusted for study size, quality or confounding and should not be read as evidence of treatment effect. The randomised trial was analysed separately.

### 2.6 Eligibility Criteria

Inclusion and exclusion criteria were defined a priori and derived from the PICO framework. Studies were eligible if they involved patients with complex extremity wounds of the hand, foot, leg or arm caused by trauma, burns or combined mechanisms; used dermal scaffolds as an early reconstructive intervention; reported outcomes relevant to skin grafting or flap reconstruction; were primary clinical studies; and fell within the publication period 2016–2026. Studies were excluded if they concerned a non‐extremity anatomical site (e.g., abdomen, breast, face or scalp) or the wrong population; if they were case reports, conference abstracts, non‐clinical studies, review articles or other secondary evidence; if no scaffold was used; if the sample comprised fewer than five patients; if they addressed scar treatment only; or if they were incomplete. Mixed populations including non‐burn or non‐trauma wound types were excluded where the relevant outcomes could not be extracted separately.

Three included studies contained a minority of participants not meeting these criteria strictly, reported here transparently: two scalp wounds among 12 patients [6]; a cohort in which 149 of 175 (85.1%) had burn aetiology, the remainder chronic wounds, infection, trauma and tumour [14]; and a mixed cohort including one diabetic foot ulcer, one oncological excision and one necrotising fasciitis [1]. These were retained because most participants were eligible and exclusion would have removed a substantial share of the evidence. A sensitivity consideration is reported in Section 3.3.

### 2.7 Risk of Bias Assessment

Risk of bias was assessed according to study design. For the single randomised controlled trial, the Cochrane Risk of Bias 2 (RoB 2) tool was used, evaluating the randomisation process, deviations from intended interventions, missing outcome data, measurement of the outcome and selection of the reported result.

For all non‐randomised studies of interventions, including retrospective cohorts, comparative observational studies and case series, the ROBINS‐I tool was used. ROBINS‐I evaluates seven domains: bias due to confounding; bias in selection of participants; bias in classification of interventions; bias due to deviations from intended interventions; bias due to missing data; bias in measurement of outcomes; and bias in selection of the reported result. The overall risk of bias for each study was classified as Low, Moderate, Serious or Critical, determined by the highest risk identified across domains. Given the predominantly retrospective nature of the literature and the absence of randomised control groups, confounding and selection bias were given particular scrutiny.

### 2.8 Assessment of Reporting and Publication Bias

Formal statistical assessment of publication bias, such as funnel plot asymmetry or Egger's test, was not appropriate because fewer than 10 studies reported a comparable effect estimate and no meta‐analytic pooling was performed. Reporting bias was therefore assessed qualitatively across three dimensions: declared financial relationships between authors and scaffold manufacturers; the balance of positive and negative findings across the identified literature; and whether prespecified outcomes appeared to have been reported selectively. Findings are reported in Section 3.9.

### 2.9 Exploratory Conceptual Framework: The Reconstructive Downstaging Index

The Reconstructive Downstaging Index (RDI) is presented in this review as an exploratory, hypothesis‐generating conceptual framework and not as an outcome measure. It was not prespecified in the review protocol, has not been validated against any external standard, has not been tested for inter‐rater or intra‐rater reliability and cannot be reproduced from the source publications without reviewer judgement. It is described here because it offers a structured vocabulary for discussing the results, not because it constitutes evidence.

The framework conceptualises reconstruction along an ordinal ladder of decreasing complexity: free flap, local flap, skin graft and secondary (spontaneous) healing. A scaffold is described as having downstaged reconstruction when a wound that would conventionally have required a more complex procedure was instead closed by a simpler method. The central and unavoidable weakness of this framework is that the conventional reconstruction is inferred by the present reviewers from wound type and anatomical site because none of the included studies reported a prespecified reconstructive plan and none included a flap comparator. Every RDI assignment therefore rests on an assumption that cannot be verified from the primary data.

Accordingly, the RDI was applied descriptively only. It was not used to derive any numerical result, was not pooled, and does not contribute to any conclusion of this review. Its application is reported separately in Section 3.12 and Table S5, and it is discussed in Section 4.6. Readers should treat it as a proposal for future investigation requiring prospective validation.

### 2.10 Deviations From the Registered Protocol

The review as conducted departs from the registered protocol in eight respects. All are declared, with justification and likely direction of effect, in Table S6, in accordance with PRISMA 2020 item 24c. None was made after inspecting the results in a manner favouring a particular conclusion.

**Two deviations are material**. First, the protocol specified no search date restriction, whereas the review was restricted to 2016–2026 for the reasons given in Section 2.1. Second, the protocol defined early application as placement within 7 days of injury or initial debridement and specified exclusion beyond that; this could not be applied because only two studies documented the interval in verifiable form. Early application was instead operationalised as application of a dermal scaffold as the primary reconstructive step, before definitive autologous coverage and a sensitivity analysis stratified by protocol compliance is reported in Section 3.3.

The remaining deviations concern the primary outcome framing, the risk of bias instruments, the addition of a reporting bias assessment, the substitution of descriptive synthesis for meta‐analysis, the retention of three studies containing a minority of protocol‐excluded participants and the number of authors. Both registered primary outcomes are reported in full and rated for certainty, so no registered outcome has been dropped.

### 2.11 Certainty of Evidence

Certainty of evidence was assessed using the GRADE approach, as specified in the registered protocol, for both registered primary outcomes, for graft avoidance stratified by aetiology, for the registered secondary outcomes of time to closure and complication rate and for scar quality, the only outcome supported by randomised evidence.

Non‐randomised evidence began at low certainty and randomised evidence at high, with downgrading for risk of bias, inconsistency, indirectness, imprecision and suspected publication bias, and no upgrading. Because no pooled estimates were generated, GRADE was applied to the direction and consistency of findings rather than to the precision of a summary estimate. Ratings were assigned independently by two reviewers.

### 2.12 Use of Artificial Intelligence

Artificial intelligence‐assisted tools were used only for language revision and consistency checking. All scientific content, including the search strategy, study selection, data extraction, synthesis, interpretation and conclusions, was generated, checked and approved by the authors.

## 3 Results

### 3.1 Study Selection and Profile

Twenty‐three full texts were assessed, 13 excluded and 10 included. Selection is shown in Figure 1; characteristics in Table 1 and Table S1.

**FIGURE 1:**
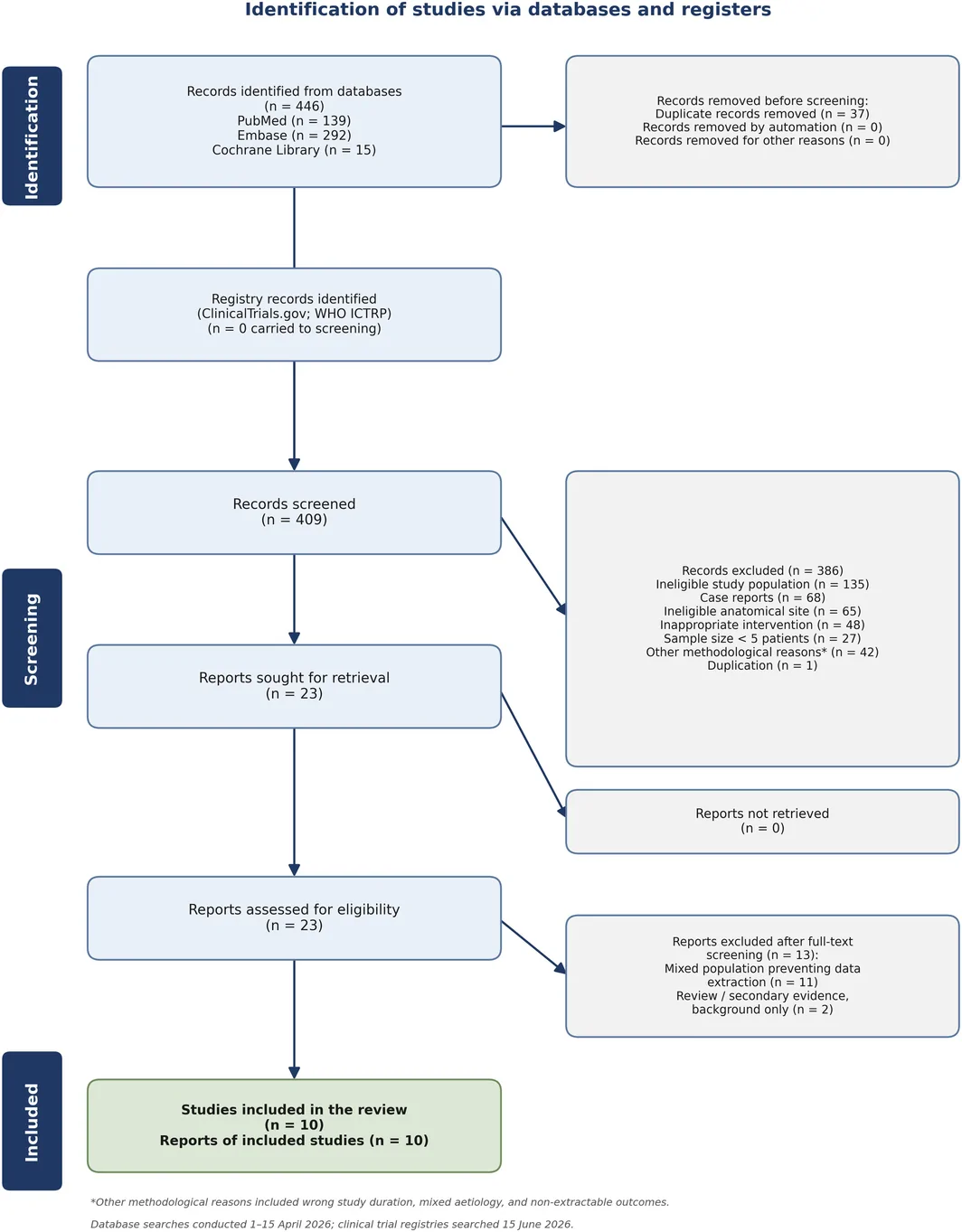
PRISMA 2020 flow diagram of study identification, screening and inclusion. The diagram follows the PRISMA 2020 statement [22].

The final dataset comprised 10 studies and 415 scaffold‐treated wounds in 414 patients: six trauma studies (192 patients), three burn studies (212 patients, including the randomised trial) and one mixed‐aetiology study (11 patients). By number of studies, trauma was the largest group; by patient numbers the burn group was largest, reflecting a single large burn cohort [14]. Nine studies were non‐randomised, contributing 393 patients; the remaining 22 patients formed the Pelnac arm of the single randomised controlled trial and were analysed separately throughout.

Designs comprised one randomised controlled trial, four retrospective comparative studies or cohorts, four case series and one prospective interventional series, representing seven scaffold products.

### 3.2 Quantitative Synthesis of Reconstructive Outcomes

Across the nine non‐randomised studies (393 patients), 38 patients (9.7%) healed without requiring skin grafting, 352 (89.6%) underwent grafting following scaffold application and 3 (0.8%) experienced treatment failure, of whom one required flap reconstruction. Per‐study and aetiology‐stratified outcomes are presented in Table 2.

**TABLE 2:** Quantitative synthesis of reconstructive and safety outcomes, by study.

Study	*n*	Graft avoidance *n* (%)	Scaffold take	Graft take	Complications *n* (%)	Secondary procedures	Failure/reconstructive escalation
Wu‐Fienberg 2021	6	3 (50.0)	Not reported (no matrix loss)	100% (no graft loss)	0 (0)	0	0
Fulchignoni 2022	27	15 (55.6)	Not reported	Not reported	Not reported	12 planned staged grafts	0
Gonzalez 2022	109	0 (0)	96.8% NPWT vs. 91.4% no NPWT (*p* = 0.20)	96.8% NPWT vs. 85.1% no NPWT (*p* = 0.03)	9 (8.2) overall; 3.2% vs. 14.9% (*p* = 0.03)	Matrix reapplication 8.2%	0
Hones 2023	11	6 (54.5)	Not reported	Complete take in both grafted patients	3 (27.3) failed to heal with matrix alone	2 delayed STSG	3 (27.3); 1 flap
Jou and Chepla 2024	27	14 (51.9)	Not reported	Closure 94.4% BTM vs. 77.8% CCS (*p* = 0.48)	BTM 6 (33.3) vs. CCS 5 (55.6)	BTM 0.67 vs. CCS 1.56 (*p* = 0.049)	2 local flaps; 1 revision amputation
Tapking 2024	175	0 (0)	82.0% ± 24.7%	87.3% ± 19.0%	47 (26.9); infection 36 (20.6)	1.6 ± 0.8 after STSG	BTM removal 15 (8.6); mortality 16 (9.1)
Song 2024	11	0 (0)	Not reported	8/11 primary healing	3 (27.3) partial edge necrosis	0 (no reoperation)	0
Yadav 2024	12	0 (0)	Not reported	10/12 (83.3) complete take	2 (16.7) SSI + partial loss	2 regrafts	0
Ling 2024	15	0 (0)	Not reported	No significant difference vs. thick STSG	No significant difference (*p* = 0.12)	Donor sites needing graft 22.2% vs. 100% (*p* < 0.001)	0
Rady 2025 (RCT)	22	0 (0)	5/22 (22.7)	Graft‐only arm 100% coverage success	Not separately reported	22 staged autografts	0 (1 death per arm, burn‐related)
Stratified summary							
Trauma (6 studies)	192	32 (16.7)	—	—	—	—	0 (0)
Burn (2 studies)	190	0 (0)	—	—	—	—	0 (0)
Mixed aetiology (1 study)	11	6 (54.5)	—	—	—	—	3 (27.3)
All non‐randomised [9]	393	38 (9.7)	—	—	—	—	3 (0.8)
Randomised trial (Pelnac arm)	22	0 (0)	—	—	—	—	0 (0)

*Note:* Values are as reported in the source publications; fields marked not reported were not stated by the original authors and were not imputed. Summary rows are unweighted crude proportions calculated from extracted event counts. They are descriptive summaries of the dataset, are unadjusted for study size, design or quality and must not be interpreted as pooled treatment effects. The randomised trial is reported separately and was not combined with non‐randomised data. Within the trauma stratum, all 32 graft‐avoidance events arose from three studies; the remaining three used protocols of immediate or planned staged grafting in which graft avoidance was not achievable.

Complete graft avoidance was observed in 32 of 192 trauma patients (16.7%) and in 0 of 190 burn patients (0%). In the mixed‐aetiology cohort, 6 of 11 patients (54.5%) healed without grafting. In the randomised trial, 0 of 22 patients in the Pelnac arm avoided grafting.

These proportions are unweighted crude counts and must not be read as pooled treatment effects (Figure 2).

**FIGURE 2:**
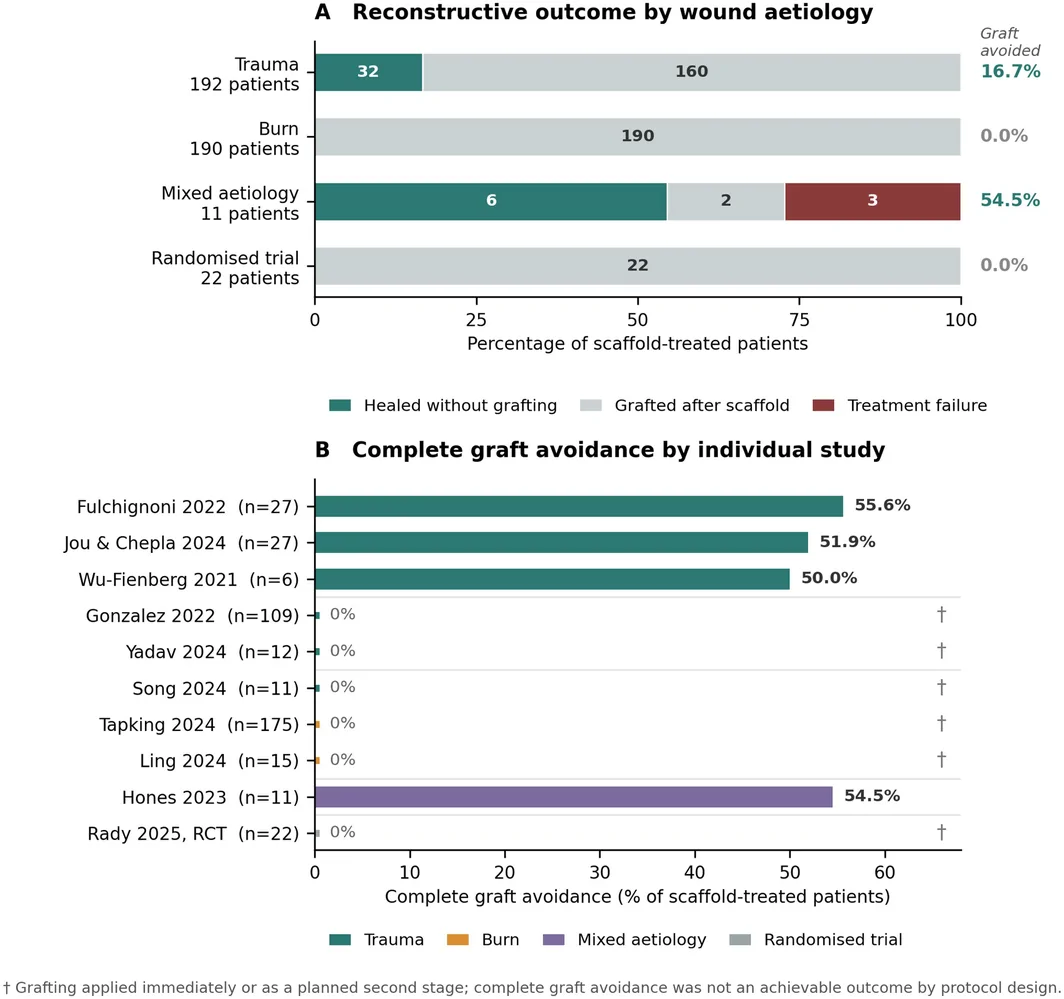
Distribution of reconstructive outcomes following dermal scaffold application, stratified by wound aetiology. Bars show the number of scaffold‐treated patients healing without grafting and undergoing grafting within the trauma, burn and mixed strata of the non‐randomised dataset (*n* = 393), with the randomised trial shown separately. Values are crude counts extracted from the source publications and are not pooled effect estimates.

### 3.3 Trauma Wounds

Six studies comprising 192 patients addressed traumatic extremity wounds.

Graft avoidance was reported by Wu‐Fienberg and colleagues in 3 of 6 upper‐extremity wounds (50.0%) [24], by Jou and Chepla in 14 of 27 upper‐extremity wounds (51.9%) [15] and by Fulchignoni and colleagues in 15 of 27 post‐traumatic fingertip defects (55.6%) [16]. In each case, the wounds in which grafting was avoided were small. Wu‐Fienberg and colleagues reported a mean defect size of 26 cm^2^ (range 10–42) in the three patients who re‐epithelialised spontaneously, compared with a series mean of 97 cm^2^ [24]. Fulchignoni and colleagues applied an explicit inclusion criterion of tissue loss below 2 cm^2^ and additionally excluded thumb injuries, degloving injuries with extensive bone exposure and injuries with associated tendon damage [16]. Jou and Chepla reported mean wound sizes in the non‐grafted subgroups of 44.8 ± 39.1 cm^2^ for BTM and 17.5 ± 3.5 cm^2^ for collagen‐chondroitin silicone [15].

By contrast, the three trauma studies reporting no graft avoidance—Gonzalez and colleagues (109 patients), Yadav and colleagues (12 patients) and Song and colleagues (11 patients)—all used protocols in which skin grafting was applied either immediately alongside the scaffold or as a planned second stage [2, 6, 7].

**Sensitivity analysis by protocol‐compliant timing**. Because the registered 7‐day definition of early application could not be applied as an eligibility criterion (Section 2.10), studies were instead stratified post hoc by whether the interval from injury or initial debridement to scaffold application was documented and met that threshold. Two studies verifiably complied: Fulchignoni and colleagues operated within 5 days of trauma [16], and Wu‐Fienberg and colleagues documented application on post‐injury Day 7 [24]. Four studies demonstrably did not: Tapking and colleagues reported a mean interval of 14.3 ± 31.9 days [14], Ling and colleagues median intervals of 17 and 22.5 days from burn to surgery [23], Song and colleagues applied the scaffold after repeated debridement of established post‐surgical complications including one wound of 18 months' duration [7], and the cohort of Hones and colleagues included an ulcer present for 9 years [1]. Four studies did not report the interval at all [2, 6, 15, 17].

Graft avoidance was 18 of 33 patients (54.5%) in the protocol‐compliant studies, 6 of 212 (2.8%) in the demonstrably non‐compliant studies and 14 of 170 (8.2%) where timing was unreported. The direction of this difference is consistent with the review's underlying premise that earlier application is associated with greater reconstructive benefit.

Both protocol‐compliant studies comprised small trauma defects—fingertip losses below 2 cm^2^ and upper‐extremity wounds averaging 26 cm^2^ in the graft‐avoiding subgroup—whereas the non‐compliant group is dominated by extensive burns. The analysis cannot separate the effect of early application from the effect of treating small, favourable wounds and it was not prespecified. It is reported because the registered protocol made timing an eligibility criterion, and silently abandoning that criterion would have been inappropriate.

Considering the two studies with eligibility deviations, a sensitivity consideration excluding the two scalp wounds from the series reported by Yadav and colleagues yields 10 extremity patients with unchanged graft avoidance (0 of 10) and one complication (10.0%). The trauma stratum denominator becomes 190 patients with an unchanged graft avoidance count of 32 (16.8%). The direction and magnitude of the finding are therefore not sensitive to this deviation.

### 3.4 Burn Wounds

Two non‐randomised studies comprising 190 patients addressed burn wounds, and no patient in either study avoided skin grafting.

Tapking and colleagues reported the largest single cohort in this review: 175 patients with at least 10% total body surface area treated surgically, of whom 149 (85.1%) had burn aetiology [14]. Mean BTM take was 82.0% ± 24.7% and mean split‐thickness skin graft take was 87.3% ± 19.0%. Earlier application to a freshly debrided bed was associated with better BTM take, whereas a greater number of surgeries prior to BTM application predicted poorer take (estimate −3.41, 95% CI −6.82 to −0.03, *p* = 0.04). Complications occurred in 47 patients (26.9%), comprising infection beneath the BTM in 36 (20.6%) and haematoma in 7 (4.0%); BTM removal was required in 15 patients (8.6%). Mean length of hospital stay was 55.1 ± 47.0 days and in‐hospital mortality was 9.1%.

Ling and colleagues compared thin split‐thickness skin grafting over acellular dermal matrix with thick split‐thickness grafting alone in deep burns of the whole hand dorsum, in a comparative cohort of 38 patients of whom 15 (18 hands) received the scaffold [23]. All patients in both groups were grafted, so the scaffold did not avoid grafting. The measured benefit was located at the donor site rather than the recipient site: the proportion of donor sites themselves requiring skin grafting was 22.2% in the scaffold group compared with 100% in the thick‐graft group (*p* < 0.001). Take rates, time to complete healing, complication rates (*p* = 0.12), total active motion (*p* = 0.22) and Jebsen‐Taylor Hand Function Test scores (*p* = 0.06) did not differ significantly. At 12 months the Vancouver Scar Scale pliability subscore was significantly better in the thick‐graft group (median 0 vs. 1, *p* = 0.01), with no differences in vascularity, pigmentation or height.

### 3.5 Mixed‐Aetiology Wounds

Hones and colleagues reported the first United States clinical experience with MatriDerm in 11 patients with complex upper‐ and lower‐extremity wounds with exposed tendon or bone, all of whom were documented as unsuitable for flap coverage on medical or social grounds [1]. Eight wounds healed: six with MatriDerm alone and two following delayed split‐thickness skin grafting. Mean time to healing in the MatriDerm‐only group was 49 days (range 7–84). Three patients did not heal with the matrix alone; one, a polytrauma patient with an open tibial fracture, subsequently underwent flap reconstruction once medically stable, representing one of three documented reconstructive escalations in the non‐randomised dataset.

Two features qualify interpretation: aetiology was heterogeneous (trauma in five, iatrogenic in two, one each of oncological excision, electrical burn, diabetic foot ulcer and necrotising fasciitis) and four patients were paediatric, aged 14 months to 12 years. The 54.5% rate should not be generalised to adults.

### 3.6 Randomised Evidence

Rady and colleagues randomised 50 patients with acute deep dermal burns of the upper limb to Pelnac followed by second‐stage autografting or to autografting alone; 22 patients per arm were analysed after losses to follow‐up and two deaths [17]. Coverage success was achieved in 100% of the graft‐only arm. Pelnac take was unsuccessful in the first 17 consecutive patients; after the protocol was revised to include more thorough debridement to fat or fascia, take succeeded in the subsequent five patients, giving an overall take rate of 5 of 22 (22.7%). All patients in the Pelnac arm ultimately underwent grafting.

Benefit was confined to scar quality and patient‐reported aesthetic satisfaction. The Vancouver Scar Scale score was significantly lower (better) in the Pelnac arm (2.40 ± 1.52 vs. 7.45 ± 1.68, *p* < 0.001), and aesthetic satisfaction was significantly higher (*p* < 0.001). There were no significant differences in the Disabilities of the Arm, Shoulder and Hand score or in patient satisfaction with functional recovery (both *p* > 0.05).

### 3.7 Flap Avoidance: Reported Versus Inferred

Each flap‐avoidance claim was classified according to its evidentiary basis (Table 3).

**TABLE 3:** Flap avoidance: Basis of claim, evidence in the source publication and factors limiting inference.

Study	Basis of claim	Evidence stated in source	Selection factors limiting inference	Assessment
Hones 2023 [1]	Author‐reported	All 11 patients documented as unsuitable for flap coverage on medical or social grounds; 8/11 healed without flap	Highly comorbid or socially complex patients; heterogeneous aetiology; 4 paediatric patients	Strongest available basis; counterfactual documented per patient. One patient nonetheless required a flap
Yadav 2024 [6]	Author‐reported	All patients achieved satisfactory closure without flap	Study explicitly excluded patients with major exposure of tendon or bone ‘which clearly required flaps’; only 2–3 cm exposure included	Author‐reported claim retained, but materially confounded by selection: the population was chosen such that flaps were not clearly indicated
Song 2024 [7]	Reviewer‐inferred	Authors discuss local and free flaps as conventional options for lateral heel defects	Small lacunar defects after calcaneal fracture complications; no comparator; *n* = 11	Reasonable clinical face validity, but not measured
Gonzalez 2022 [2]	Reviewer‐inferred	No flap comparator; study compared NPWT versus no NPWT	Wounds 2–30 cm^2^ (median 7 cm^2^), predominantly forearm; such defects would not routinely mandate flap reconstruction	Inference not supported. No flap avoidance is claimed for this cohort in the present revision
Wu‐Fienberg 2021 [24]	Reviewer‐inferred	Authors describe BTM as a potential alternative to flap reconstruction in selected patients	*n* = 6; graft‐avoiding subgroup mean 26 cm^2^; author is a paid consultant for the manufacturer	Author framing is permissive, not a measured outcome

*Note:* Two distinct claims are separated in this table and should not be conflated. The first is whether the original authors reported that flap reconstruction was avoided; the second is whether the present reviewers inferred that a flap would have been the conventional alternative. For Yadav and colleagues the first holds—the authors did report closure without flap—while the second has been withdrawn, and Table S5 accordingly records skin graft, not flap, as the inferred conventional reconstruction for that cohort. For Gonzalez and colleagues neither claim is supported. No included study contained a flap comparator group, so in no case can it be established that the patients treated would otherwise have undergone flap reconstruction. Reconstructive escalation to a flap occurred in 3 patients across the dataset.

**No included study contained a flap comparator group**. Consequently, in no study can it be established that the patients treated with a dermal scaffold would otherwise have undergone flap reconstruction.

Flap avoidance was documented by the original authors in two studies. Hones and colleagues stated that all 11 patients were assessed as unsuitable for flap coverage, providing a counterfactual at the level of individual clinical decision‐making, although one patient nonetheless progressed to a flap [1]. Yadav and colleagues reported closure without flap in all patients [6].

The claim in the latter study is, however, materially confounded by its selection criteria: Yadav and colleagues explicitly excluded patients with major exposure of tendon and bone ‘which clearly required flaps’, and included only patients with minor exposure measuring 2–3 cm [6]. The study population was therefore selected on the basis that flap reconstruction was not clearly indicated, and the finding that flaps were avoided is in part a consequence of that selection.

In three further studies—Song and colleagues [7], Gonzalez and colleagues [2] and Wu‐Fienberg and colleagues [24]—flap avoidance was inferred by the present reviewers rather than reported by the original authors, and is now identified as such (Table 3). The inference retains reasonable face validity for lateral heel defects, where the original authors themselves discuss local and free flaps as the conventional alternative [7], and is permissive but unmeasured for the upper‐extremity BTM series, whose authors describe the matrix as a potential alternative to flap reconstruction in selected patients [24]. It is weakest for Gonzalez and colleagues, whose wounds ranged from 2 to 30 cm^2^ (median 7 cm^2^), lay predominantly on the forearm and were compared for negative pressure wound therapy versus none rather than scaffold versus flap [2]; defects of that size and site would not routinely mandate a flap, and the inference is not supported.

In addition, reconstructive escalation was documented in three patients across the dataset: one flap in the series reported by Hones and colleagues [1], and one local flap in the BTM arm together with one local flap and one revision amputation in the collagen‐chondroitin silicone arm reported by Jou and Chepla [15].

### 3.8 Intervention and Protocol Heterogeneity

The seven products differ in composition, architecture, degradation profile and staging; characteristics are tabulated in Table S2.

The two products associated with graft avoidance, MatriDerm and NovoSorb BTM, both support spontaneous epithelialisation across a thin construct, whereas DuraGen and human acellular dermal matrix were used exclusively within immediate‐grafting protocols and so could not demonstrate graft avoidance. No product appeared in more than three studies and none was compared against no scaffold in the same anatomical setting; stratification by product was therefore not feasible, and the aetiology‐stratified analysis confounds product effect with protocol and indication.

Stratified by protocol rather than by product or aetiology, graft avoidance occurred in 38 of 71 wounds (53.5%) in the four studies permitting observation before grafting [1, 15, 16, 24], and in none of 344 wounds in the six that grafted immediately or as a planned second stage [2, 6, 7, 14, 17, 23]. All three burn studies fall in the latter group.

### 3.9 Reporting and Publication Bias

Three of the 10 included studies had declared financial relationships with scaffold manufacturers, and all three reported findings favourable to the manufacturer's product. The senior author of both Wu‐Fienberg and colleagues [24] and Jou and Chepla [15] is a paid consultant for PolyNovo, the manufacturer of NovoSorb BTM; both studies reported outcomes favouring BTM. A co‐author of Gonzalez and colleagues [2] is an employee of Integra LifeSciences, whose Meshed Bilayer Wound Matrix was the intervention studied. Notably, the two studies with PolyNovo consultancy relationships also contributed 17 of the 38 graft‐avoidance events (44.7%) in the entire non‐randomised dataset.

Six of the remaining seven declared no conflict of interest; Song and colleagues contained no such statement. Five declared no external funding [2, 6, 14, 16, 17]; Ling and colleagues [23] had public research grants.

No study reported an overall negative or neutral conclusion, despite the randomised trial showing inferior early coverage success and Ling and colleagues showing better scar pliability in the control arm. No formal test of small‐study effects was possible.

### 3.10 Risk of Bias

#### 3.10.1 Non‐Randomised Studies

Nine of the 10 included studies were non‐randomised and were assessed using ROBINS‐I (Table S3). The non‐randomised evidence base demonstrated a high risk of bias overall, with all nine studies classified as Serious or Critical. Studies lacking any control group or relying on opportunistic case selection—Hones and colleagues, Wu‐Fienberg and colleagues and Song and colleagues—were judged to carry a Critical overall risk. The remaining comparative retrospective cohorts and observational series were judged Serious. The predominant limitations lay in confounding (Domain 1) and participant selection (Domain 2), driven by retrospective design, non‐randomised comparison and absence of adjusted analysis. Absence of blinded outcome assessment (Domain 6) contributed consistently across studies (Figure 3).

**FIGURE 3:**
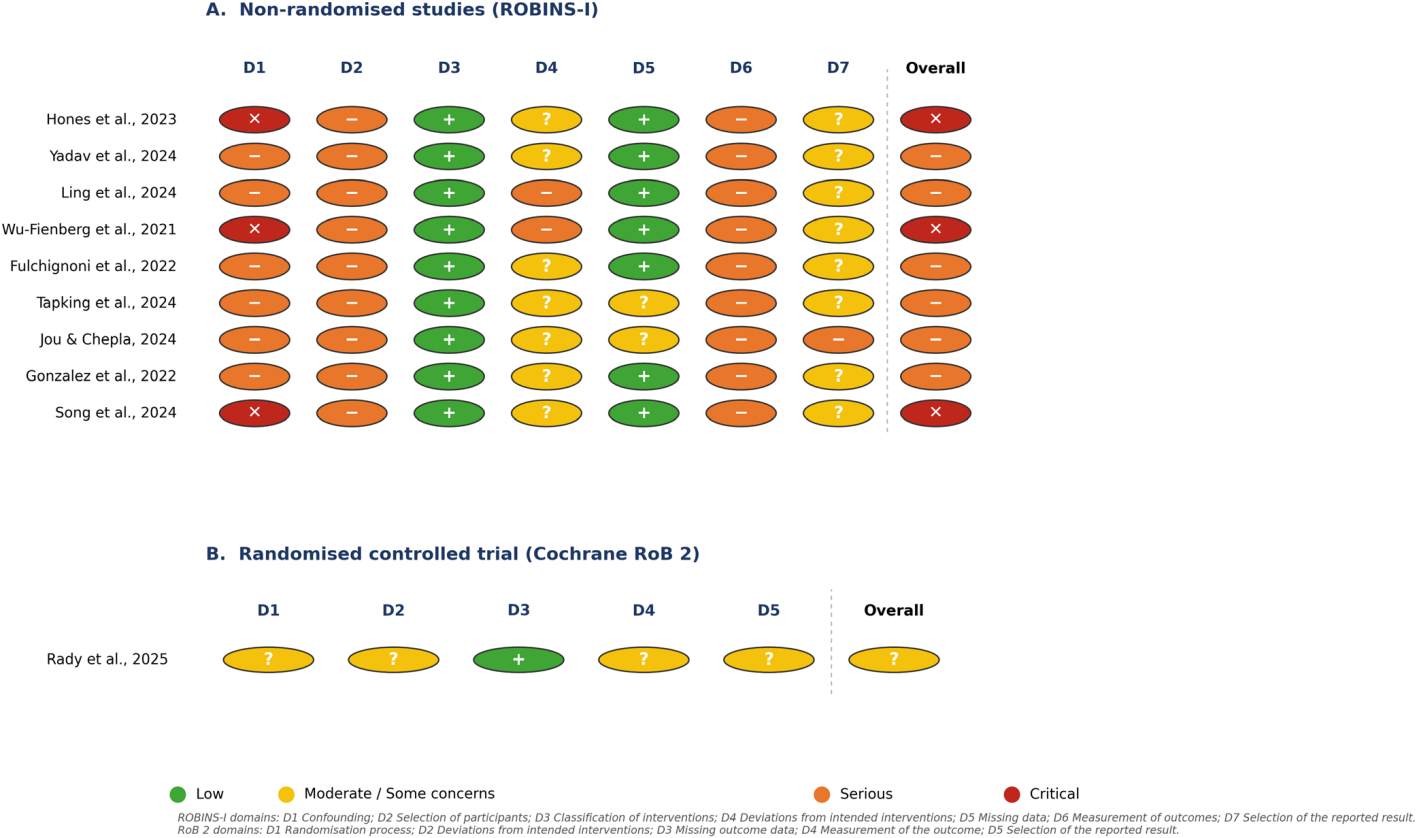
Risk‐of‐bias summary (traffic‐light plot). Panel (A) shows the nine non‐randomised studies assessed with ROBINS‐I; Panel (B) shows the single randomised controlled trial assessed with the Cochrane RoB 2 tool. Domain keys are provided beneath the figure.

#### 3.10.2 Randomised Controlled Trial

The trial by Rady and colleagues was assessed using RoB 2 (Table S4) and received an overall judgement of some concerns. Risk of bias due to missing outcome data was low, whereas the randomisation process, deviations from intended interventions, measurement of the outcome and selection of the reported result each raised some concerns. These ratings reflect limited reporting of the randomisation procedure, absence of blinding during clinical assessment and the mid‐study protocol change in debridement depth, which represents a deviation from the intended intervention that materially affected the primary outcome. No domain was rated high risk.

### 3.11 Certainty of the Evidence

Certainty ratings are presented in Table 4. Of seven outcomes, five were very low and two low; none reached moderate or high. Both registered primary outcomes were very low.

**TABLE 4:** GRADE assessment of the certainty of the evidence.

Outcome	Studies contributing	Starting certainty	Risk of bias	Inconsistency	Indirectness	Imprecision	Publication bias	Final certainty and principal reason
Requirement for flap reconstruction (registered primary)	10 studies (415 wounds)	Low	−1 Serious	Not serious	−1 Serious	Not serious	−1 Suspected	VERY LOW—no study contained a flap comparator; outcome partly inferred
Skin graft take rate (registered primary)	8 studies (366 wounds)	Low	−1 Serious	−1 Serious	Not serious	Not serious	−1 Suspected	VERY LOW—no standardised definition; assessment unblinded in all studies
Complete graft avoidance—trauma	6 studies (192 wounds)	Low	−1 Serious	−1 Serious	−1 Serious	Not serious	−1 Suspected	VERY LOW—rates 0%–55.6%; highly selected small wounds; 17/38 events from manufacturer‐linked studies
Complete graft avoidance—burns	2 studies (190 patients)	Low	−1 Serious	Not serious	Not serious	Not serious	Not serious	LOW—consistent across both studies; 0 events in 190 patients
Time to definitive wound closure	6 studies (245 wounds)	Low	−1 Serious	−1 Serious	Not serious	Not serious	Not serious	VERY LOW—37.5–106.3 days; no common definition of closure
Complication rate	8 studies (366 wounds)	Low	−1 Serious	−1 Serious	Not serious	Not serious	−1 Suspected	VERY LOW—0%–26.9%; no standard classification applied
Scar quality (Vancouver Scar Scale)	1 RCT (44 patients)	High	−1 Some concerns	Not serious	Not serious	−1 Serious	Not serious	LOW—single trial; mid‐study protocol change; small sample

*Note:* Bodies of evidence composed of non‐randomised studies began at low certainty and randomised evidence at high certainty, in accordance with GRADE guidance. No outcome was upgraded, as none demonstrated a large effect with adequate control of confounding. Because no pooled effect estimates were generated (Section 2.5), imprecision was judged on event counts and sample size rather than on the width of a confidence interval, and GRADE was applied to the direction and consistency of the extracted findings; this is a limitation of the assessment and is noted in Section 4.8. Publication bias was rated as suspected where an outcome drew materially on studies with declared manufacturer relationships (Section 3.9).

Abbreviation: RCT, randomised controlled trial.

Absence of graft avoidance in burns was rated low certainty, the highest awarded to any non‐randomised outcome, being consistent across both studies with zero events in 190 patients. Improvement in scar quality was also rated low, downgraded from high because it rests on a single randomised trial with some concerns and imprecision. Graft avoidance in trauma was rated very low, downgraded for inconsistency (0%–55.6%), indirectness from highly selected small wounds and publication bias, since 17 of 38 events derive from studies with manufacturer relationships. The claims this review can make with least uncertainty are therefore its negative and qualifying ones, and the conclusions are framed accordingly.

### 3.12 Exploratory Application of the Reconstructive Downstaging Index

The following mapping is exploratory and is presented for hypothesis generation only. It is not an outcome of this review and contributes to none of its conclusions. Every assignment of an inferred conventional reconstruction rests on reviewer judgement that could not be verified against the source data because no included study reported a prespecified reconstructive plan or contained a flap comparator.

Within these constraints, the dominant pattern across the dataset corresponded to a shift of approximately one step towards simpler closure. In extensive burns, the observed pathway was most consistent with conversion of a non‐graftable bed into a graftable one. In selected small trauma, fingertip and exposed‐structure defects, the observed pathway was most consistent with conversion of a graft‐level wound into secondary healing. Complete elimination of reconstruction was uncommon and confined to small, carefully selected wounds. In one patient, reconstruction was escalated rather than simplified. The mapping is presented in Table S5.

## 4 Discussion

### 4.1 Principal Findings

Across 10 studies and 415 scaffold‐treated wounds, complete avoidance of skin grafting following early dermal scaffold application was uncommon, occurring in 38 of 393 non‐randomised patients (9.7%), and was confined almost entirely to traumatic wounds. No burn patient in this dataset avoided grafting. The predominant reported role of dermal scaffolds was preparation of a wound bed for subsequent grafting rather than replacement of grafting.

Current evidence therefore suggests dermal scaffolds may reduce reconstructive burden in selected patients, most clearly in small traumatic defects with exposed structures, whereas in burns their contribution lies in enabling staged coverage and improving scar quality rather than reducing the number of procedures. Because the evidence is predominantly retrospective, uncontrolled and at serious to critical risk of bias, these are provisional interpretations rather than conclusions about treatment effect.

The two outcomes rated with greatest certainty are both qualifying rather than supportive: the absence of graft avoidance in burns and the improvement in scar quality. The claims this review can make with least uncertainty are therefore its negative ones, while the more encouraging trauma findings rest on the weakest evidence.

### 4.2 Trauma Reconstruction

The trauma evidence is the more encouraging of the two aetiological groups, but it is also the more susceptible to selection effects. The three studies reporting graft avoidance did so at rates between 50.0% and 55.6%, which is clinically substantial. However, the wounds concerned were consistently small: below 2 cm^2^ by protocol in one study, a mean of 26 cm^2^ in the spontaneously healing subgroup of another and predominantly under 45 cm^2^ in the third. These are wounds at the smaller end of the spectrum that prompts consideration of dermal scaffolds in the first place, and several would have been candidates for conservative management or simple grafting irrespective of scaffold use.

The three trauma studies reporting no graft avoidance were not failures of the intervention but studies in which grafting was part of the intended protocol from the outset. Reporting an aggregate trauma graft‐avoidance rate without this qualification would be misleading, and the distinction is made explicit in Table 2. The practical implication for surgeons is that graft avoidance appears achievable in small upper‐extremity and fingertip defects when the surgical plan deliberately allows for a period of observation before committing to grafting, and that this possibility is foreclosed by protocols applying immediate grafting.

Comparative evidence within the trauma group is limited to a single scaffold‐versus‐scaffold comparison. Jou and Chepla found that BTM required grafting less frequently than collagen‐chondroitin silicone (44.4% vs. 55.6%), with fewer complications (33.3% vs. 55.6%), fewer secondary procedures (0.67 vs. 1.56, *p* = 0.049) and lower total reconstruction cost [15]. Successful wound closure did not differ significantly (94.4% vs. 77.8%, *p* = 0.48), and time to grafting was longer with BTM (43.4 vs. 21.4 days, *p* = 0.002). The trade‐off is therefore between fewer procedures and a longer treatment course. It should also be noted that the reported *p* value for the difference in grafting frequency is stated as 0.013 in the abstract and 0.027 in the results of the source publication.

### 4.3 Burn Reconstruction

The burn evidence supports a different and more limited role. In 190 non‐randomised burn patients and 22 randomised patients, not one avoided skin grafting. This should not be read as evidence that burn wounds cannot heal without grafting after scaffold application, since no included burn study permitted observation before grafting. Dermal scaffolds in burns functioned as a staged dermal platform enabling grafting when immediate grafting was not feasible, and this is the role that should be communicated to patients and to commissioners.

The safety profile in extensive burns deserves emphasis because it has not featured prominently in previous syntheses. In the largest cohort, infection beneath the matrix occurred in 20.6% of patients, the matrix required removal in 8.6%, overall complications reached 26.9%, mean length of stay was 55.1 days and in‐hospital mortality was 9.1% [14]. Although mortality in this population reflects burn severity rather than scaffold use, these figures situate scaffold reconstruction within a high‐risk clinical context and argue against framing dermal scaffolds as a low‐morbidity alternative in extensive burns.

The one measurable benefit in burns identified in this review was located at the donor site. Ling and colleagues demonstrated that thin grafting over acellular dermal matrix reduced the proportion of donor sites themselves requiring grafting from 100% to 22.2% (*p* < 0.001), while achieving comparable healing and hand function [23]. In patients with limited donor reserve, this is a meaningful advantage, and it is more precisely described as donor‐site burden reduction than as graft‐burden reduction. Set against it, scar pliability at 12 months was marginally better in the thick‐graft control arm (*p* = 0.01).

The randomised evidence reinforces this cautious reading. In the only randomised comparison, graft‐only treatment achieved 100% coverage success against a Pelnac take rate of 22.7%, and all Pelnac patients were nonetheless grafted [17]. The benefit was confined to scar quality, where it was substantial and statistically robust (Vancouver Scar Scale 2.40 vs. 7.45, *p* < 0.001). Taken together with the meta‐analysis of van den Bosch and colleagues published in this journal, which found improved scar quality but delayed wound closure with dermal substitutes in burns [19], the present findings are consistent with a scaffold role in burns defined by tissue quality rather than reconstructive simplification.

### 4.4 Heterogeneity of the Interventions

Pooling seven products under a single heading risks obscuring clinically important differences, and this review does not claim a class effect. As set out in Section 3.8, the pattern observed is plausibly explained in part by construct thickness and intended staging rather than by biological superiority.

The aetiology‐stratified analysis should therefore be read as descriptive of the available literature rather than as isolating a biological effect of any individual scaffold. Head‐to‐head comparison of scaffolds within a single indication remains a substantial evidence gap; only one such comparison exists in this dataset [15].

### 4.5 Cost and Economic Considerations

Given the acquisition cost of dermal scaffolds, economic evaluation is central to their adoption, yet only one included study addressed cost directly. Jou and Chepla reported institutional 2017 prices of USD 850 per 100 cm^2^ for NovoSorb BTM against USD 3150 per 100 cm^2^ for Integra collagen‐chondroitin silicone [15]. Mean template placement cost did not differ significantly (USD 865.50 vs. USD 2040.50, *p* = 0.075), but total reconstruction cost including secondary procedures was significantly lower with BTM (USD 1361.92 vs. USD 3185.71, *p* = 0.049), driven principally by fewer secondary operations (0.67 vs. 1.56). When length of stay was incorporated at USD 3226 per hospital day, the difference was no longer significant (USD 7276.25 vs. USD 13,222.15, *p* = 0.11). Yadav and colleagues noted that DuraGen cost less than half the price of Integra at their institution, but performed no formal analysis [6].

Several limitations constrain these data. No study performed a cost‐utility analysis, reported incremental cost‐effectiveness ratios or quality‐adjusted life years or adopted a societal perspective. Costs derive from a single United States institution using Medicare fee schedules and are not transferable. Critically, no study compared scaffold‐based against flap reconstruction—the comparison most relevant to this review. Economic evaluation remains an open question rather than a settled advantage.

### 4.6 Interpreting the Reconstructive Downstaging Framework

This framework is offered as a way of organising discussion, not as a finding (Figure 4). The Reconstructive Downstaging Index has not been validated, has not been tested for reproducibility, was not prespecified and cannot be derived from the source publications without reviewer inference. It should not be used to guide clinical decisions and no conclusion of this review depends upon it.

**FIGURE 4:**
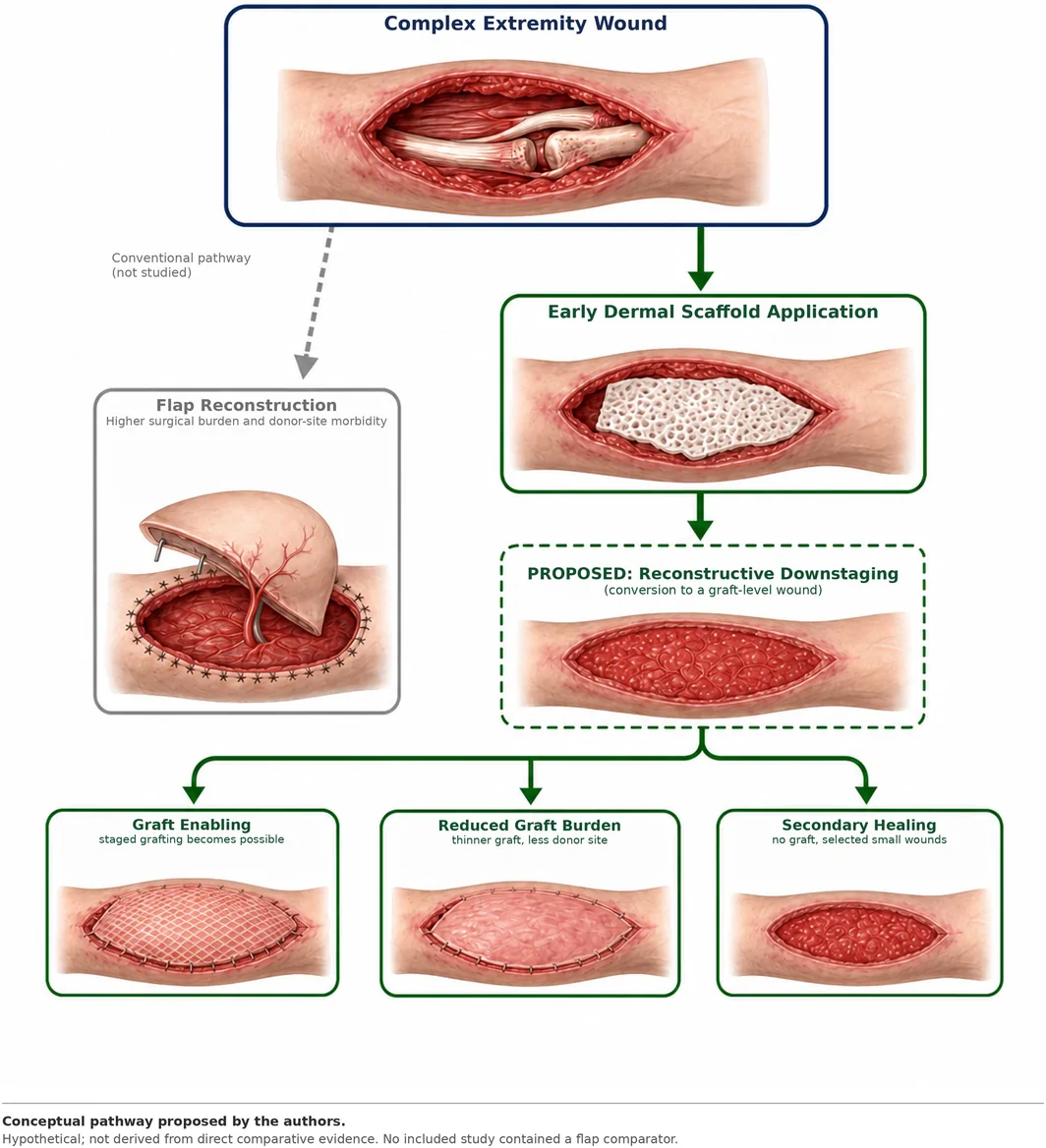
Proposed conceptual pathway of reconstructive downstaging. This figure is hypothetical and represents the authors' synthesis of the included literature; it is not derived from direct comparative evidence, and no included study contained a flap comparator or reported a prespecified reconstructive plan. Complex extremity wounds with exposed tendon, bone or joint structures conventionally shift reconstruction towards local or free flap surgery. The figure illustrates a proposed alternative pathway in which early dermal scaffold application generates a vascularised wound bed, with three possible consequences: graft enabling (preparing an initially non‐graftable wound for later grafting), graft‐burden or donor‐site burden reduction and, in selected small defects, healing without grafting. The pathway requires prospective validation before it can be used to guide clinical decisions.

Its potential value is conceptual. Existing outcome measures record whether a wound healed and capture poorly whether the reconstruction required became simpler. A framework describing movement along the reconstructive ladder [25] could, if operationalised and validated prospectively, provide a common language for interventions whose benefit lies in procedural simplification. That would require prospective recording of intended reconstruction, blinded adjudication and formal reliability testing—none of which the present evidence meets.

### 4.7 Clinical Implications

#### 4.7.1 Trauma

In selected traumatic wounds—small upper‐extremity, hand, foot or fingertip defects with exposed tendon or bone—dermal scaffolds may permit closure without flap reconstruction and, in a subset of cases, without grafting. Where the wound is small and the surgical plan permits a period of observation before committing to grafting, graft avoidance appears achievable in approximately half of such cases in reported series, although these series are small and highly selected. Where the plan involves immediate grafting, the realistic benefit is coverage over exposed structures rather than avoidance of a procedure.

#### 4.7.2 Burns

In burn wounds, dermal scaffolds should not be presented to patients as a means of avoiding grafting. No patient in this review's burn dataset avoided grafting. Their role is to provide a vascularised dermal platform enabling delayed grafting when immediate grafting is not feasible, to reduce donor‐site requirement in patients with limited donor reserve and to improve scar quality. Performance is strongly dependent on debridement adequacy and on prompt application to a freshly debrided bed, and the complication and infection rates in extensive burns are not trivial.

#### 4.7.3 Common Considerations

Across both populations, wound‐bed preparation was the dominant technical determinant of success: Pelnac take improved from 0 of 17 to 5 of 5 after deeper debridement [17]; BTM take fell with each prior procedure [14]; and adjunctive negative pressure wound therapy improved graft take from 85.1% to 96.8% while reducing complications from 14.9% to 3.2% [2]. Scaffold selection should remain individualised, balancing physiological reserve, donor‐site limitation, comorbidity and the morbidity of flap reconstruction against a substantial acquisition cost.

### 4.8 Limitations

This review has several important limitations, which together mean that its findings should be regarded as hypothesis‐generating.

First, and most importantly, flap avoidance was inferred rather than measured. No included study contained a flap comparator, and in no case can it be established that a patient treated with a dermal scaffold would otherwise have required flap reconstruction. Two studies documented flap avoidance at the level of individual clinical decision‐making; in three further studies the inference was made by the present reviewers and is explicitly identified as such in Table 3. In one of these, the study population had been selected specifically to exclude patients who clearly required flaps, which confounds the claim directly.

The inference is weakest for the largest of these cohorts, in which wounds ranged from 2 to 30 cm^2^ with a median of 7 cm^2^ and lay predominantly on the forearm, and the study comparator was negative pressure wound therapy rather than flap reconstruction; that inference is not supported and has been withdrawn.

Second, outcome definitions and units were not standardised: no consistent definition of graft avoidance, flap avoidance, scaffold take or complication was used, scaffold take was assessed unblinded, follow‐up ranged from 3 to 12 months, and denominators varied between fingers, hands and patients. Fifteen of the 38 graft‐avoidance events are finger‐level, so pooled proportions describe wounds rather than individual patients.

Third, publication and sponsorship bias is likely present: three of 10 studies had declared manufacturer relationships, all reported favourable findings and together they contributed 44.7% of graft‐avoidance events. No study reached a wholly negative conclusion. Formal assessment of small‐study effects was not possible.

The absence of any wholly negative report across a literature dominated by small retrospective series is itself consistent with selective publication of favourable experience.

Fourth, meta‐analysis was not feasible: seven distinct products, no comparator in seven of 10 studies, non‐standardised outcomes and marked variation in wound size and site made pooling inappropriate. Reported proportions are unweighted crude counts.

Fifth, selection bias limits generalisability: most cohorts comprised small wounds treated at experienced reconstructive or specialist burn centres (median 7 cm^2^ in one, below 2 cm^2^ by protocol in another, 26 cm^2^ in the graft‐avoiding subgroup of a third), and eight of 10 studies enrolled fewer than 30 patients. Findings should not be extrapolated to large defects, non‐specialist settings or unselected populations.

The apparent association between protocol‐compliant timing and graft avoidance reported in Section 3.3 is similarly confounded, since both protocol‐compliant studies comprised small trauma defects while the non‐compliant group is dominated by extensive burns; timing cannot be separated from wound size or aetiology in these data.

Sixth, three studies deviated from the eligibility criteria in a minority of participants, as set out in Section 2.6; a sensitivity consideration in Section 3.3 shows the principal finding unchanged. Four paediatric patients were included within one cohort.

Seventh, the search window of 2016–2026 excludes historically important studies of dermal substitutes, as set out in Section 2.1. The review therefore describes contemporary rather than complete evidence.

Eighth, the Reconstructive Downstaging Index is unvalidated, unprespecified and dependent on reviewer inference, as discussed in Sections 2.9 and 4.6.

Ninth, the review deviates from its registered protocol in several respects, most materially in the search date restriction and in the operationalisation of early application, both of which are declared with justification in Section 2.10 and Table S6 and accompanied by sensitivity analyses. Certainty assessment by GRADE was necessarily applied to the direction and consistency of findings rather than to the precision of pooled estimates because no pooled estimates were generated.

### 4.9 Future Research

Prospective multicentre studies are required, ideally randomised or prospectively matched, using standardised endpoints agreed across burn and trauma communities.

Endpoints should include: the reconstruction intended before scaffold application, documented by an independent blinded surgeon, so that flap avoidance becomes measured rather than inferred; number and type of subsequent procedures; donor‐site area and morbidity; scaffold and graft take by blinded adjudication against a defined threshold; time to definitive closure; complication and reoperation rates by standard classification; validated patient‐reported and site‐appropriate functional measures; scar quality by Vancouver Scar Scale or POSAS at 12 months; and cost‐effectiveness analysis comparing scaffold against flap reconstruction.

Two further priorities follow from this review. Head‐to‐head comparison of scaffold products within a single indication is almost absent, with one exception and is needed before any class effect can be claimed. And if the concept of reconstructive downstaging is to be more than descriptive, it requires prospective operationalisation and formal reliability testing before it can be used as an outcome measure.

## 5 Conclusion

This systematic review of 10 studies and 415 scaffold‐treated wounds found that early dermal scaffold application was associated with complete avoidance of skin grafting in a minority of patients (9.7% of the non‐randomised dataset), and that this benefit differed fundamentally between aetiologies. Separate conclusions are therefore warranted.

In traumatic extremity wounds, current evidence suggests that dermal scaffolds may permit closure without grafting in approximately half of carefully selected small defects, and may allow coverage of exposed tendon or bone without recourse to flap reconstruction in patients for whom flap surgery is undesirable. This potential appears confined to small wounds treated with protocols that permit observation before committing to grafting, is derived from series of fewer than 30 patients at serious to critical risk of bias, and has never been tested against a flap comparator.

In burn wounds, dermal scaffolds should not be regarded as a means of avoiding autologous grafting; no burn patient in this dataset did so, although no burn study permitted a period of observation before grafting, so this reflects protocol design as well as any biological limitation. Their demonstrated contributions are provision of a vascularised platform enabling delayed grafting, reduction of donor‐site requirement in patients with limited donor reserve, and improvement in scar quality. The only randomised evidence available showed inferior early coverage success compared with grafting alone, with benefit confined to scar quality.

Across both populations, the value of dermal scaffolds appears to lie in simplifying reconstruction rather than eliminating it, and their use is most readily justified in patients with exposed tendon or bone, limited flap options, high comorbidity burden or a need to minimise donor‐site morbidity. Success is strongly dependent on adequate debridement and wound‐bed preparation. The concept of reconstructive downstaging is offered as an exploratory framework for future investigation and not as a validated outcome.

Stronger comparative studies with prospectively recorded reconstructive intent, standardised outcome definitions, longer follow‐up and formal economic evaluation are required before firm practice recommendations can be made.

## Funding

The authors have nothing to report.

## Ethics Statement

The authors have nothing to report.

## Conflicts of Interest

The authors declare no conflicts of interest.

## Supporting information


**Table S1:** Included primary clinical studies in the systematic review. This table lists the primary clinical studies included and summarises study design, scaffold used, scaffold‐treated sample, wound context and the main reconstructive role relevant to the synthesis.
**Table S2:** Characteristics of the dermal scaffold products represented in the included studies.
**Table S3:** Risk of bias in the nine non‐randomised studies (ROBINS‐I).
**Table S4:** Risk of bias in the single randomised controlled trial (Cochrane RoB 2).
**Table S5:** Exploratory conceptual mapping of study outcomes onto the reconstructive ladder (Reconstructive Downstaging Index).
**Table S6:** Deviations from the registered PROSPERO protocol (CRD420261360486, version 2.0).

**Figure S1:** The scaffold spectrum. This matrix categorises the dermal scaffolds evaluated according to material composition (synthetic vs. biological or biosynthetic) and the reconstructive role observed in the included studies, ranging from graft avoidance through graft‐burden reduction and graft enabling to improvement in healing or scar quality alone. Wound aetiology associated with each product is indicated. The figure illustrates that the observed role of a scaffold in this dataset is confounded with wound type, defect size and treatment protocol, and should not be read as a comparative statement of product performance.

## Data Availability

All data supporting this systematic review were extracted from previously published studies cited in this article and are summarised in the manuscript and Supporting Information. No new primary datasets were generated.
