# Supplementary material for: Dermal Scaffolds in Complex Extremity Wound Reconstruction: Impact of Early Application on Grafting and Flap Requirements—A Systematic Review
Source: Wound Repair Regen. 2026 Oct 4;34(5):e70219. doi: 10.1111/wrr.70219 (PMC13634600; doi:10.1111/wrr.70219)
Supplement: Supplementary file 1 [file WRR-34-0-s002.docx]

**Supporting Information**

*Dermal Scaffolds in Complex Extremity Wound Reconstruction: Impact of Early Application on Grafting and Flap Requirements — A Systematic Review (manuscript 9789965)*

**Supplementary Table S1.** Included primary clinical studies in the systematic review. This table lists the primary clinical studies included and summarises study design, scaffold used, scaffold-treated sample, wound context, and the main reconstructive role relevant to the synthesis. STSG, split-thickness skin graft; BTM, biodegradable temporising matrix; ADM, acellular dermal matrix; HADM, human acellular dermal matrix; NPWT, negative pressure wound therapy; CCS, collagen-chondroitin silicone bilayer matrix; IMBWM, Integra Meshed Bilayer Wound Matrix.

| **No.** | **Study** | **Journal** | **Study design** | **Wound aetiology / site** | **Scaffold / intervention** | **Sample included in synthesis** | **Main reconstructive role / key outcome** |
| --- | --- | --- | --- | --- | --- | --- | --- |
| 1 | Wu-Fienberg et al., 2021 | Plastic and Reconstructive Surgery Global Open | Retrospective case series | Complex upper-extremity wounds with exposed tendon and/or bone | NovoSorb BTM | 6 scaffold-treated patients | Trauma-focused reconstructive downstaging. Three patients re-epithelialised without grafting and three underwent delayed STSG; no infections or matrix/graft loss reported. |
| 2 | Fulchignoni et al., 2022 | Journal of Cosmetic Dermatology | Retrospective clinical study | Post-traumatic fingertip tissue loss | MatriDerm | 27 fingers / 26 patients | Graft avoidance in selected small fingertip defects. Fifteen fingers healed with MatriDerm alone and 12 required delayed grafting; functional, aesthetic, and sensibility outcomes were comparable between groups. |
| 3 | Gonzalez et al., 2022 | Journal of Wound Care | Retrospective comparative study | Complex extremity wounds, predominantly trauma, with exposed structures | Integra Meshed Bilayer Wound Matrix with or without NPWT | 109 patients | Graft-enabling dermal matrix reconstruction. NPWT improved STSG take and reduced reapplications and postoperative complications compared with IMBWM alone. |
| 4 | Hones et al., 2023 | Journal of Wound Care | Retrospective case series | Complex upper- and lower-extremity wounds with exposed tendon and/or bone in patients unsuitable for flap reconstruction | MatriDerm | 11 patients | Flap-sparing/downstaging role. Eight wounds healed successfully: six with MatriDerm alone and two after delayed STSG; three cases did not fully heal with matrix alone. |
| 5 | Jou and Chepla, 2024 | ePlasty | Retrospective comparative cost analysis | Isolated traumatic upper-extremity soft-tissue defects | NovoSorb BTM versus Integra CCS | 27 patients (18 BTM, 9 CCS) | Cost and reconstructive burden reduction. BTM required fewer secondary procedures, fewer complications, and less frequent skin grafting than CCS. |
| 6 | Tapking et al., 2024 | Journal of Clinical Medicine | Retrospective cohort study | Extensive burn wounds (>10% TBSA surgically treated) | NovoSorb BTM followed by STSG or Meek grafting | 175 patients | Graft-enabling role in extensive burns. Mean BTM take was 82.0% and mean STSG take was 87.3%; earlier application after debridement was associated with better BTM take. |
| 7 | Song et al., 2024 | Advances in Skin & Wound Care | Case series | Lacunar lateral heel soft-tissue defects after calcaneal fracture complications | HADM combined with STSG and NPWT | 11 patients | Graft-enabling and defect-filling role for heel defects. All wounds healed; three had partial edge necrosis but healed with wound care and no reoperation. |
| 8 | Yadav et al., 2024 | Tropical Doctor | Prospective interventional case series | Suboptimal wounds with minor exposed tendon or bone, mainly post-traumatic extremity wounds | DuraGen with immediate split skin graft | 12 patients | Flap avoidance for limited exposed structures. Ten patients had complete graft uptake; two had partial loss/SSI but all achieved final wound closure without flap reconstruction. |
| 9 | Ling et al., 2024 | International Wound Journal | Retrospective comparative cohort study | Deep/full-thickness burns of the whole dorsum of the hand | Thin STSG over ADM versus thick STSG | 38 patients; 15 scaffold-treated patients / 18 hands | Graft-burden and donor-site morbidity reduction. ADM + thin STSG achieved similar healing and function to thick STSG, with markedly lower donor-site grafting requirement. |
| 10 | Rady et al., 2025 | European Journal of Plastic Surgery | Randomized controlled trial | Acute deep dermal burns of the upper limb | Pelnac dermal substitute followed by second-stage autografting versus graft-only control | 22 Pelnac patients analysed | Scar-quality and staged coverage role, but not reliable graft replacement. Pelnac take was successful in 5/22 after more thorough debridement to fat/fascia; successful Pelnac cases had improved VSS and aesthetic satisfaction compared with graft-only patients. |

**Supplementary Table S2.** Characteristics of the dermal scaffold products represented in the included studies.

| **Product** | **Class** | **Composition** | **Layers** | **Staging** | **Studies (n)** | **Reconstructive role observed in this review** |
| --- | --- | --- | --- | --- | --- | --- |
| NovoSorb BTM | Synthetic | Biodegradable polyurethane foam with non-biodegradable sealing layer | Bilayer | Two-stage (3–4 weeks) | 3 | Graft avoidance in small trauma defects; graft-enabling in burns |
| Integra CCS / Meshed Bilayer | Biosynthetic xenogenic | Bovine collagen cross-linked with shark chondroitin-6-sulphate under silicone | Bilayer | Two-stage (2–3 weeks) | 2 | Graft-enabling; comparator in cost analysis |
| MatriDerm | Biological xenogenic | Native bovine collagen types I, III, V with bovine elastin, not cross-linked | Single layer | Single-stage or spontaneous epithelialisation | 2 | Graft avoidance in fingertip and flap-unsuitable wounds |
| PELNAC | Biosynthetic xenogenic | Porcine tendon collagen sponge with silicone film | Bilayer | Two-stage | 1 | Scar-quality improvement only |
| Human ADM | Biological allogenic | Decellularised human cadaveric dermis | Single layer | Single-stage with graft | 1 | Graft-enabling; defect filling |
| ADM (allogenic) | Biological allogenic | Decellularised dermis beneath thin split-thickness graft | Single layer | Single-stage composite | 1 | Donor-site burden reduction |
| DuraGen | Biological xenogenic | Bovine collagen dural substitute, used off-label | Single layer, porous | Single-stage with immediate graft | 1 | Graft-enabling over minor exposed structures |

Products differ in composition, thickness, degradation profile, and intended staging. Products applied within immediate-grafting protocols could not, by design, demonstrate graft avoidance. No product was represented by more than three studies and no study compared a scaffold against no scaffold in the same anatomical setting; stratification by product was therefore not feasible. ADM, acellular dermal matrix; BTM, biodegradable temporising matrix; CCS, collagen-chondroitin silicone.

**Supplementary Table S3.** Risk of bias in the nine non-randomised studies (ROBINS-I).

| **Study** | **D1** | **D2** | **D3** | **D4** | **D5** | **D6** | **D7** | **Overall** |
| --- | --- | --- | --- | --- | --- | --- | --- | --- |
| Hones 2023 | Critical | Serious | Low | Moderate | Low | Serious | Moderate | Critical |
| Yadav 2024 | Serious | Serious | Low | Moderate | Low | Serious | Moderate | Serious |
| Ling 2024 | Serious | Serious | Low | Serious | Low | Serious | Moderate | Serious |
| Wu-Fienberg 2021 | Critical | Serious | Low | Serious | Low | Serious | Moderate | Critical |
| Fulchignoni 2022 | Serious | Serious | Low | Moderate | Low | Serious | Moderate | Serious |
| Tapking 2024 | Serious | Serious | Low | Moderate | Moderate | Serious | Moderate | Serious |
| Jou & Chepla 2024 | Serious | Serious | Low | Moderate | Moderate | Serious | Serious | Serious |
| Gonzalez 2022 | Serious | Serious | Low | Moderate | Low | Serious | Moderate | Serious |
| Song 2024 | Critical | Serious | Low | Moderate | Low | Serious | Moderate | Critical |

D1, confounding; D2, selection of participants; D3, classification of interventions; D4, deviations from intended interventions; D5, missing data; D6, measurement of outcomes; D7, selection of the reported result. Studies lacking a control group or using opportunistic case selection without adjustment for major covariates were rated Serious or Critical in D1 and D2, giving a Serious or Critical overall judgement, as expected for retrospective case series and cohorts.

**Supplementary Table S4.** Risk of bias in the single randomised controlled trial (Cochrane RoB 2).

| **Study** | **Randomisation process** | **Deviations from intended interventions** | **Missing outcome data** | **Measurement of the outcome** | **Selection of the reported result** | **Overall** |
| --- | --- | --- | --- | --- | --- | --- |
| Rady 2025 (17) | Some concerns | Some concerns | Low risk | Some concerns | Some concerns | Some concerns |

Ratings reflect limited reporting of the randomisation procedure, absence of blinding during clinical assessment, and a mid-study protocol change to deeper debridement that materially altered the primary outcome. No domain was rated high risk.

**Supplementary Table S5.** Exploratory conceptual mapping of study outcomes onto the reconstructive ladder (Reconstructive Downstaging Index).

**This table is exploratory and hypothesis-generating. It is not an outcome of this review and supports none of its conclusions.**

| **Study** | **Scaffold** | **Wound type / site** | **Inferred conventional reconstruction*** | **Reconstruction observed after scaffold** | **Direction of movement*** |
| --- | --- | --- | --- | --- | --- |
| Wu-Fienberg 2021 | NovoSorb BTM | Trauma, upper extremity | Skin graft | Secondary healing 3/6; graft 3/6 | Graft → secondary healing (↓1) in ~50% |
| Fulchignoni 2022 | MatriDerm | Trauma, fingertip <2 cm² | Skin graft or conservative management | Healed without graft 15/27; delayed graft 12/27 | Graft → secondary healing (↓1) in ~56% |
| Jou & Chepla 2024 | NovoSorb BTM vs Integra CCS | Trauma, upper extremity | Skin graft (standard template) | Reduced graft requirement, fewer secondary procedures | Graft-burden reduction (no ladder step) |
| Tapking 2024 | NovoSorb BTM | Burn, ≥10% TBSA | Non-graftable bed / staged coverage | Staged graft after scaffold integration | Non-graftable → graft-level (↓1) |
| Song 2024 | Human ADM | Post-traumatic, lateral heel | Local or free flap (author-discussed) | Scaffold + graft | Flap → graft (↓1), inferred |
| Yadav 2024 | DuraGen | Trauma, minor exposed structures | Skin graft (flaps excluded by design) | Scaffold + immediate graft | No ladder step; prior flap inference withdrawn |
| Gonzalez 2022 | Integra Meshed Bilayer | Trauma, extremity 2–30 cm² | Skin graft | Scaffold + staged graft | No ladder step; prior flap inference withdrawn |
| Ling 2024 | ADM | Burn, hand dorsum | Thick split-thickness graft | ADM + thin graft; donor-site requirement reduced | Donor-site burden reduction (no ladder step) |
| Hones 2023 | MatriDerm | Mixed, extremity; flap-unsuitable | Flap (documented as unsuitable) | Healed without graft 6/11; delayed graft 2/11; 3 failures | Flap → secondary healing (↓2) in 6/11; 1 escalation (↑1) |
| Rady 2025 (RCT) | PELNAC | Burn, upper limb | Skin graft | Graft in all; improved scar quality | No ladder step; quality benefit only |

*The inferred conventional reconstruction reflects the reconstruction the wound would typically have required without a dermal scaffold, inferred by the present reviewers from wound type and site rather than from any within-study comparator or prespecified plan. No included study reported an intended reconstruction or contained a flap comparator, so no assignment in this table can be verified against the primary data. ↓ indicates movement towards simpler closure; ↑ indicates escalation. Two flap inferences present in the original submission (Gonzalez 2022, Yadav 2024) have been withdrawn following re-examination of the source publications. ADM, acellular dermal matrix; BTM, biodegradable temporising matrix; CCS, collagen-chondroitin silicone; RCT, randomised controlled trial; TBSA, total body surface area.

**Supplementary Figure S1.** The scaffold spectrum. This matrix categorises the dermal scaffolds evaluated according to material composition (synthetic versus biological or biosynthetic) and the reconstructive role observed in the included studies, ranging from graft avoidance through graft-burden reduction and graft enabling to improvement in healing or scar quality alone. Wound aetiology associated with each product is indicated. The figure illustrates that the observed role of a scaffold in this dataset is confounded with wound type, defect size, and treatment protocol, and should not be read as a comparative statement of product performance.

**Supplementary Table S6.** Deviations from the registered PROSPERO protocol (CRD420261360486, version 2.0).

| **Protocol element** | **As registered** | **As conducted** | **Justification and likely direction of effect** |
| --- | --- | --- | --- |
| Search dates | No search date restrictions | Restricted to 2016–2026 | Corresponds to the period in which current-generation scaffolds entered routine extremity practice (§2.1). Excludes historically important reports; narrows the evidence base and limits comparison with earlier literature |
| Definition of early application | Within 7 days of injury or initial debridement; later application excluded unless reported separately | Application as the primary reconstructive step, before definitive autologous coverage | Only two studies documented the interval in verifiable form. Sensitivity analysis by protocol compliance reported in §3.3; retaining non-compliant studies is conservative with respect to the review's premise |
| Primary outcomes | Requirement for flap reconstruction; skin graft take rate | Graft burden (cancellation, sparing, flap avoidance) | Both registered primary outcomes are reported in full (§3.7, Table 3, Table 2) and rated in Table 4. The registered outcome set was broadened, not replaced |
| Risk of bias instruments | ROBINS-E and MINORS | ROBINS-I and Cochrane RoB 2 | ROBINS-E addresses exposures rather than interventions; MINORS cannot accommodate a randomised trial. Change expected to yield a more conservative appraisal |
| Reporting bias | Will not be assessed | Qualitative assessment of reporting and sponsorship bias performed (§2.8, §3.9) | Added following peer review. An addition to the registered methods; produced findings unfavourable to the intervention |
| Data synthesis | Meta-analysis, random-effects, I² heterogeneity | Descriptive synthesis without pooling | Registered fallback to narrative synthesis invoked, for the reasons in §2.5. I² not calculable in the absence of pooled estimates |
| Population exclusions | Diabetic, pressure and venous ulcers; oncologic and post-surgical wounds excluded | Three studies retained containing a minority of such participants | Majority of participants in each study were eligible; exclusion would have removed a substantial share of the evidence. Affected participants identified in Table 1; sensitivity consideration in §3.3 |
| Review team | Two members | Five authors | Additional authors contributed to interpretation, clinical framing and revision. All meet ICMJE criteria |
| Title | Reconstructive Downstaging Following Early Dermal Scaffold Application in Complex Extremity Wounds: A Systematic Review (current PROSPERO record, version 2.0) | Dermal Scaffolds in Complex Extremity Wound Reconstruction: Impact of Early Application on Grafting and Flap Requirements — A Systematic Review | The manuscript carries the title under which the manuscript is registered with the journal (manuscript 9789965), which is also the title under which the review was originally registered on PROSPERO (record version 1.0, 7 April 2026). The version 2.0 title was judged on reflection to presuppose the effect under investigation and was not retained following peer review. Both titles are retrievable from the public PROSPERO record through the “Show differences with previous version” view |

Deviations are declared in accordance with PRISMA 2020 item 24c. None was made after inspection of the results in a manner favouring a particular conclusion.
